# The enigma of aluminum deposition in bone tissue from a patient with chronic kidney disease: a case report

**DOI:** 10.1590/2175-8239-JBN-3882

**Published:** 2018-06-04

**Authors:** Rodrigo Dias de Meira, Cinthia Esbrile Moraes Carbonara, Kélcia Rosana da Silva Quadros, Carolina Urbini dos Santos, Patrícia Schincariol, Gustavo de Souza Pêssoa, Marco Aurélio Zezzi Arruda, Vanda Jorgetti, Rodrigo Bueno de Oliveira

**Affiliations:** 1 Universidade Estadual de Campinas, Faculdade de Ciências Médicas, Departamento de Medicina Interna, Campinas, SP, Brasil.; 2 Universidade Estadual de Campinas, Departamento de Medicina Interna (Nefrologia) - Faculdade de Ciências Médicas, Laboratório para o Estudo do Distúrbio Mineral e Ósseo em Nefrologia (LEMON), Campinas, SP, Brasil.; 3 Universidade Estadual de Campinas, Instituto de Química, Grupo de Espectrometria, Preparo de Amostras e Mecanização, Departamento de Química Analítica, Campinas, SP, Brasil.; 4 Universidade de São Paulo, Departamento de Medicina Interna, São Paulo, SP, Brasil.

**Keywords:** Kidney Failure, Chronic, Dialysis, Bone Diseases, Metabolic, Aluminum, Doença Renal Crônica, Diálise, Doenças Ósseas Metabólicas, Alumínio

## Abstract

About four decades ago, the relationship between dialysis-dementia and aluminum (Al) began to be established. The restriction of drugs containing Al and improvements on water quality used for dialysis resulted in the clinical disappearance of Al intoxication. However, high prevalence of Al deposition in bone tissue from Brazilian dialysis patients is still being detected. Through the case report of a patient on hemodialysis (HD) for one year, presenting significant Al deposition in bone tissue, we speculated if this problem is not being underestimated. We used extensive investigation to identify potential sources of Al exposure with a careful review of medication history and water quality controls. Al concentration was measured by different methods, including mass spectrometry, in poly-electrolyte concentrate solutions and solution for peritoneal dialysis, in an attempt to elucidate the possible sources of contamination. The objective of this case report is to alert the medical community about a potential high prevalence of Al deposition in bone tissue and to discuss the possible sources of contamination in patients with chronic kidney disease (CKD).

## RESUMO

Cerca de quatro décadas atrás, a relação entre demência relacionada à diálise e alumínio (Al) começou a ser estabelecida. A restrição de medicamentos contendo Al e melhorias na qualidade da água utilizada na diálise resultaram no desaparecimento clínico da intoxicação por Al. Contudo, no Brasil continua a ser identificada uma elevada prevalência de deposição de Al no tecido ósseo de pacientes em diálise. O presente relato de caso de um paciente em hemodiálise (HD) há um ano com deposição significativa de Al no tecido ósseo nos leva a especular se esse problema não tem sido subestimado. Realizamos uma ampla investigação para identificar possíveis fontes de exposição ao Al, com uma revisão cuidadosa do histórico de medicação e dos controles de qualidade da água. A concentração de Al foi medida por diferentes métodos, incluindo espectrometria de massa, nos concentrados polieletrolíticos para hemodiálise e soluções de diálise peritoneal, na tentativa de elucidar as possíveis fontes de contaminação. O objetivo do presente relato de caso é alertar a comunidade médica sobre uma possível elevada prevalência de deposição de Al no tecido ósseo e discutir as possíveis fontes de contaminação nos pacientes com doença renal crônica (DRC).

## INTRODUCTION

Aluminum (Al) is the most abundant metal on earth and human beings are often exposed to it.^1^ The accumulation and toxicity of this metal was noted in hemodialysis (HD) patients in the 1970's, and osteomalacia, anemia, and dementia were associated with exposure to water, dialysate preparations, or drugs containing Al.^2^^-^ 4 Since improvements on water treatment were established and the use of non-Al-containing phosphate (P) binders became standard practice, the prevalence of Al intoxication with clinical signs almost disappeared.^4^^-^ 5 Therefore, it was assumed that Al-related bone diseases would also have disappeared. This potential misconception was supported by clinical and serum Al levels evaluations only, instead of the gold standard method: bone biopsy stained by solochrome azurine.

Brazil is one of the countries with the largest number of dialysis patients in the world and has about 700 dialysis units. Most units use reverse osmosis for water treatment, and quality requirements are similar to the European and American guidelines, being controlled under Federal legislation.^6^^-^ 7 Four laboratories in Brazil are specialized on renal osteodystrophy and perform bone histomorphometric analysis and histological studies for Al detection. These centers have an accumulated experience of more than 5,000 bone biopsies from chronic kidney disease (CKD) patients. Recently, the Brazilian Registry of Bone Biopsy (REBRABO) was created as a research platform on this field.^8^ Data analysis has detected a high prevalence of Al deposition in bone samples from Brazilian CKD patients over the decades.^9^^,^ 10 Therefore, we claim attention to potential under-diagnosis of Al deposition in bone tissue in other countries as well.

We present the case of a patient who had been on HD for just one year and was diagnosed with Al deposition in bone tissue. An extensive investigation was carried out to identify potential sources of Al exposure.

## CASE REPORT

A 36-year-old man with CKD of undetermined etiology started peritoneal dialysis (PD). After 3 years, he switched to HD due to an episode of fungal peritonitis. He remained clinically stable during the first year of HD and never presented any signs or symptoms related to mineral and bone metabolism disorders, such as bone pain, pruritus, muscular weakness, pathological fracture, signs of vascular calcification or neurological symptoms. His physical examination was normal. Overtime he developed asymptomatic hyperparathyroidism, presenting serum intact parathyroid (iPTH) levels of 467 pg/mL, P of 3.8 mg/dL, calcium (Ca) of 9.5 mg/dL, alkaline phosphatase (AP) of 92 IU/L, and Al of 13 mcg/L [methodology: graphite furnace-atomic absorption spectrometry (GFAAS); reference range: < 30 mcg/L].^7^

**Figure 1:**
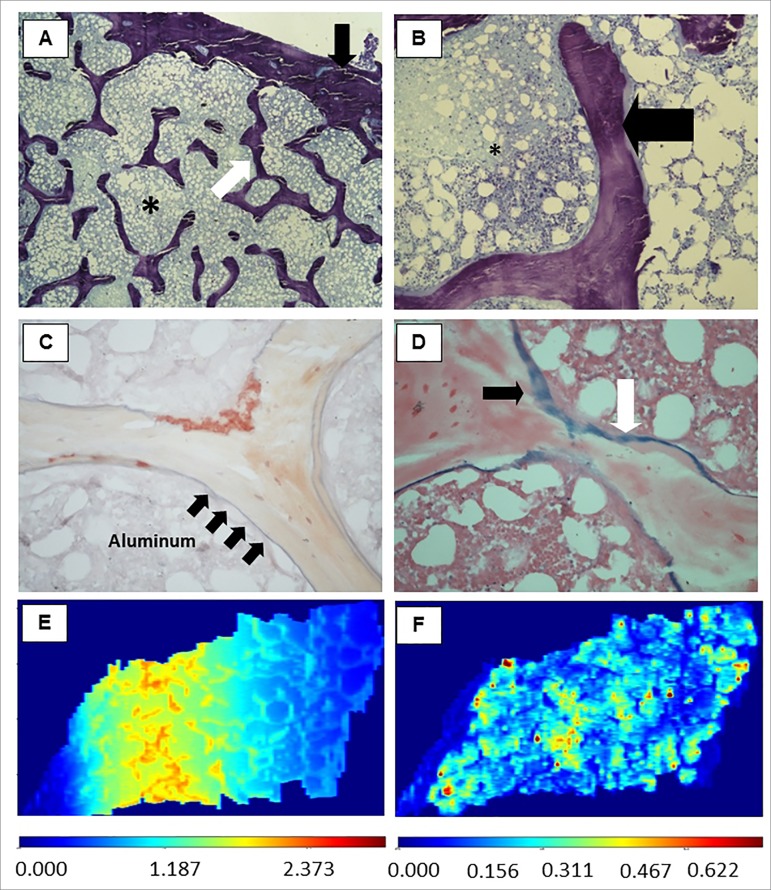
Representative images of bone tissue. (A) Thin cortical and trabecular bone with an increased trabecular separation (x40 magnification). (B) Trabecular bone with osteoid (x400). (C) Solochrome azurine staining revealing in blue (black arrows) the aluminum deposition in bone interface (mineralization front) along almost the entire trabeculae (x400); (D) Pearls staining revealing deposits of iron predominantly in trabecular bone. *bone marrow; black arrows: cortical bone; white arrows: trabecular bone; black broad arrow, osteoid; arrows: black, Al deposition in Figure 1C and iron deposition in Figure 1D. Images of bone tissue showing the deposition of Al and Fe, constructed by LA-iMageS software with data obtained from the analysis of LA-ICP-MS. (E) Distribution of Al predominantly in trabecular bone tissue; (F) Distribution of Fe predominantly in bone marrow. Sidebars refer to the intensity of the elements present in the tissue: high intensity (dark red and red) or low intensity (dark blue and blue), in-between: average intensity.

At this moment, the patient was included in a clinical study, and a transiliac bone biopsy was performed. The sample obtained consisted of two cortical and trabecular bone samples revealing the diagnosis of osteitis fibrosa. Unexpectedly, the coloration of solochrome azurine was positive for Al, covering 50% of the bone surface.^11^^-^ 14 Pearls' staining was positive for iron in a similar extent (Figure 1A to 1D). Treatment with desferoxamine at 5 mg/kg once a week for 6 months was initiated, with follow-up exams revealing serum levels of Ca 10.2 mg/dL, P 2.2 mg/dL, iPTH 263 pg/mL, AP 47 IU/mL, and Al 4.7 mcg/L. At the end of the treatment, the patient was still asymptomatic and without signs of Al intoxication or bone disease. One year after being submitted to bone biopsy the patient underwent renal transplantation.

The unexpected diagnosis of Al deposition has led to the investigation of sources of exposure, such as medications, water for HD, polyelectrolyte concentrates, and PD solution bags. Review of medical records has shown the patient had never used antacids, Al-based P binders, or any medications that could deliberately contain Al. In the last 3 years, he had never presented alterations in annual serum Al levels (GFAAS, reference range: < 30 mcg/L).^7^ Al detection analyses in HD water treated by reverse osmosis provided negative results (two samples, separated by one year) (methodology: inductively-coupled plasma optical emission spectrometry; reference range < 10 µg/L).^7^

We tested bone tissue samples, water used in the dialysis unit, polyelectrolyte concentrate solutions, and PD solution bags using inductively-coupled plasma mass spectrometry (ICP-MS) with laser ablation (LA) techniques. The chemical elements present in the sample were ionized by high plasma temperature. Only ions Fe^+^ and Al^+^ were selected, generating a signal proportional to their quantities in the samples. The technique is based on the use of a laser for ablating the sample, and the vapor generated in the process is transported by an inert gas (argon) to the inductively coupled plasma torch. LA-ICP-MS lecture can be converted to an imaging mode containing the distribution of metal in the tissue.^15^^-^ 16 This qualitative analysis was performed on bone tissue using the LA-ICP-MS technique, through a Perkin-Elmer brand equipment (DRC-e model) and a LA unit (New Wave-UP213). The images were treated with the software LA-iMageS.^16^ Using a slide obtained from the same fragment of bone tissue, the presence of Al and Fe deposits was confirmed, with clear discrimination between them (Figure 1 E-F).

Samples of water (N = 4), polyelectrolyte concentrate solutions (N = 5; two different trademarks), and PD solution bags (N = 1), were normalized with the addition of a standard concentration of 50 µg/L of Al. The accuracy of the method was evaluated using the certified reference material of trace elements in natural waters (SRM 1640A), obtaining a value of 52.9 ± 1.2 µg/L, compared with the certified value of 52.6 ± 1.8 µg/L. The results show that all analyzed samples by means of the ICP-MS method were negative for Al (Table 1).

**Table 1:** Quantification of Aluminum by ICP-MS in different water samples and solutions used in dialysis unit. The concentration of Al in all samples was very close to the value of the normalization concentration added to each sample

Samples	Al concentration (µg/L)
PCHD (acid) trademark A	46.7 ± 0.8
PCHD (acid) trademark B (sample 1)	50.2 ± 0.9
PCHD (acid) trademark B (sample 2)	50.3 ± 1.5
SCB trademark A (sample 1)	46.9 ± 0.5
SCB trademark A (sample 2)	47.6 ± 0.5
Peritoneal dialysis solution trademark C	50.4 ± 0.7
Reverse osmosis outlet water (sample 1)	51.5 ± 0.6
Reverse osmosis outlet water (sample 2)	51.5 ± 0.7
Pre-treatment inflow water (sample 1)	51.5 ± 1.0
Pre-treatment inflow water (sample 2)	49.2 ± 0.5
Dialysate at the input of the HD machine	49.7 ± 0.5

HD: hemodialysis; PCHD: polyelectrolyte concentrate for hemodialysis; SCB: sodium bicarbonate concentrate; Al: aluminum.

## DISCUSSION

Al intoxication in dialysis patients with classic signs and symptoms of Al-encephalopathy and osteomalacia has ceased to be considered a clinical problem for several years and is rather considered a rare event.^2^^,^ 3 However, deposition of Al in bone tissue, especially in the mineralization front ("bone intoxication by Al") has a high prevalence in Brazil; a multicenter study found 2,507 bone biopsies from patients with clinical, radiological, or laboratory indications of bone disease. A prevalence of Al intoxication was 61.3% between 1985-1990, 38.7% between 1991-1996, and 42.5% between 1997-2001.^9^ A survey in 2008 from data of the REBRABO study revealed a prevalence of Al intoxication of 42% in 149 samples.^8^^,^ 10

Therefore, we believe that Al intoxication is still an important problem in Brazil and perhaps in other countries. We hypothesize that its clinical manifestation is currently attenuated, with potential repercussions on anemia and bone disease. Al causes a decrease in heme synthesis and interferes with iron metabolism leading to microcytic anemia. Rao et al. studied 18 HD patients under erythropoietin (EPO) treatment and observed a trend for poor EPO-response in those with high deposition of Al in osteoid surfaces.^17^ The accumulation of this metal in bone tissue causes osteomalacia and adynamic bone disease. These effects are mediated through interferences on parathyroid hormone synthesis and release. Studies have reported Al deposition in parathyroid glands and disturbances of calcium-sensing-receptor activity.^18^^,^ 19

However, discriminating the consequences or symptoms of Al toxicity can be difficult because they are usually nonspecific and are present in several diseases that affect patients with CKD. Related symptoms are proximal muscle weakness, bone pain, spontaneous fractures, acute alteration in mental status, and premature osteoporosis. It should be noted that serum Al levels are not reliable markers of organ deposition and bone biopsy is the definitive approach for the diagnosis of Al-related bone disease. 

Two other possibilities for Al contamination are from medicine and food. Medications for patients undergoing dialysis may contain Al, especially in intravenous form, such as dipyrone, erythropoietin, and iron sulfate.^20^ The impact of this contamination is unknown. As for diet, data on intestinal absorption of Al in healthy subjects reveal that small quantities (0.06 - 0.1%) are absorbed from food sources. Factors that may influence absorption and its bioavailability are compounds that bind to Al in the intestinal lumen, gastric acidity, and hardness of water consumed.^21^ Patients with celiac disease may have increased intestinal permeability to Al, and can thus develop Al-related bone disease.^22^ None of these conditions was observed in our patient.

Unfortunately, we did not evaluate Al content in the ingested water and intravenous drugs used by the patient. We believe that the main source of Al exposure for CKD patients is the water used for dialysis, although we could not prove this. The ICP-MS could be a differential and complementary technique for a frequent evaluation of fluids and drugs used in the treatment of these patients, aiming to avoid exposure to Al. Additionally, its complementary technique (LA-ICP-MS) can discriminate safely which metal is deposited in the tissue. In this case report a limited amount of samples was analyzed, while the patient had contact with 360 L or more of water per week for years. We cannot affirm that polyelectrolyte concentrates and PD solution bags were not sources of contamination, since only a few samples were analyzed.

## CONCLUSION

Al intoxication may be largely under-diagnosed, perhaps in several regions of the world. There is an urgent need for clinical studies with bone biopsy in this field in order to confirm our hypothesis. Considering that doses of Al in fluids have limited diagnostic value and bone biopsy is an invasive procedure and restricted to a few centers, both ICP-MS and LA-ICP-MS are promising techniques that can be used to understand the phenomenon of Al intoxication in patients on dialysis, helping in the identification of contamination sources. Systemic Al intoxication is an unusual event nowadays, but deposition of Al in bone tissue can be a frequent event, which can cause important clinical outcomes, such as fractures and death.
